# The Country Dentist

**Published:** 1911-04-15

**Authors:** W. E. Green

**Affiliations:** Kirksville, Mo.


### THE COUNTRY DENTIST.

W. E. GREEN, KIRKSVILLE, MO.

There has been much written during the past two years relative to the dentist, his income, his savings, his term of service, how to get more business, etc., until one would think that we were all on the road to the poor house.

The articles to which I have especial reference are: Business Building and Brother Bill's Letters in the *Dental Digest*, and Extending the Field of Dentistry in the *Dental Brief*.

These articles seem to have been written for some other locality, or for the city dentist. In the latter case I have nothing to say, for I know nothing of city ways nor of city dentists. Yet in the cities there seems to be a demand for more work, in that the dentists are undertaking to educate the public to the value of dental services.

Before discussing the country dentist I would like to make what seems to me a plain statement of facts. Wealth is comparative. An income that permits a man to live as comfortably as the average of the better class of people in his community has, in my mind, a competence and is comparatively well to do. This amount will vary for different communities. An income of $800 in one locality will compare with $1,200 in another or $3,000 in still another.

It is presumed that a dentist is a man of limited means and must earn his way in life. If possessed of the unusual financial ability of a Rockefeller or some other man of high attainments, he would not be looking for just a good living and freedom from want in his old age, so would not turn to dentistry.

It seems to me that in country towns the best salaried men are the heads of the banks. In the first place they are men of business experience, also men of means to invest to secure their positions. While it would seem hardly fair to compare a dentist with a banker, yet I believe that some dentist in every town has as large a salary as the heads of the local banks.

Next for merchants. Upon inquiry I find that a merchant expects to have sales two and one-half ($2\frac{1}{2}$) times his capital stock, and is fairly well pleased with a ten per cent profit on his gross sales, this ten per cent to include his salary and interest upon his investment. That is, $20,000 invested should bring $50,000 sales, at ten per cent gives $5,000 for salary and interest.

According to the journals it will cost a young man $5,000 in expense of education, loss of time and cost of fitting up an office. Five thousand dollars in capital invested at two and one-half equals $12,500 at ten per cent will give $1.250 for salary and interest.

Now will a dentist in a town that will support a $20,000 store make $1,250? I think so, and enough more to give good interest on the $5,000.

The dentist carries most of his investment in his head. Thieves cannot break in and steal it, fire destroy it, nor should age depreciate it. After a time, it is true, his income is not so great, but he earns something as long as he visits his office.

I believe every man is entitled to succeed, and does so to the extent of his ability, unless by some foolish act, bad habit, or presumption he drives success from him. We, as dentists, do not always attain the success we wish, so some discussion of the cause for this will not come amiss.

In the first place we usually think we are entering the millionaire class, due partly to the fact that as soon as we announce to our friends that we are going to become a dentist, we are told what a fine business it is and how much money is made by some dentist of our acquaintance. So we begin to look upon life from that angle and begin to live accordingly. As soon as we are out of school we begin to keep up appearances by good clothes and a good office, which are necessary; but also the unnecessary acts of trying to be a liberal spender, trying to keep up with some old practitioner, not knowing that perhaps his credit is so low that he cannot get supplies without remitting with the order.

We try to gain business by "treats" to those whose business we want, also by cutting the price or giving some part of the service. Why do we do this? Has it ever occurred to us that the other fellow is in a position to go us one better if he so desired? When the clothier comes in we throw in an extra filling for his influence. Does he ever throw in anything but a pair of suspenders to keep our trousers up when we buy a suit? Does our grocer throw in an extra sack of flour or bushel of potatoes when we settle our bill? So on down the line. Every man with whom we have mercantile dealings needs our business more than we need theirs.

Oh! it is the influence? If influence can be bought on any basis other than good service we do not want it, for the other fellow can buy it the same way.

Now, how about that shrewdest of bargainers, the farmer? Does he give you nine feet of wood for a cord, or the extra pounds of corn over the even bushels? Certainly not; you pay for everything you get all the way around.

Now, as a side light upon this throwing in something for good measure, do you happen to know how much the service actually cost you? Many dentists don't. From actual accounting in my office expenses for the last fourteen years I have found the year's business to vary from twenty five to thirty-five per cent. This does not take into account the refurnishing of the office, which I have done twice in the last ten years, and now I have hardly anything of the old equipment of ten years ago. So let us take thirty per cent as an average cost of running an office. If you were to hire a man on a per cent basis you would have to guarantee him a certain amount of business, carry the credit and then pay him at least fifty per cent for all work he would do for you. So allowing yourself the fifty per cent, without guarantee, etc., and add thirty per cent actual expense, your operations cost you eighty per cent.

In an ordinary country practice I find that patients will average $5.00 each, so with a $2,000 practice you will have 400 patients. Now give each service amounting to $1.00, and you give away $400 in business, which cost you, at eighty per cent, $320 each year. How do you like your generosity? That money would buy you a home or educate a child in the course of your years of service.

It seems to me that we need to look to the fifty per cent salary for our living and savings, for the twenty per cent profit will not much more than take care of bad accounts, depreciation in office and our repair work. Our business may be increased by applying plain principles of honesty. Give none but honest service. If the patient can't afford the highest priced work, give them good, honest work of the cheaper grade. Talk for high class work, the kind you know to be best for their case. You will frequently be surprised at the result from showing a patient how much more beneficial a bridge will be than the plate that they had contemplated. Make your office handy by proper arrangement, and also by getting equipment. You may think that you cannot afford it, but you cannot afford to do without it. Also you will find that with years of experience you will be able to turn out more good, honest work.

Give the best service every time, or nothing. Get a profit; let the other fellow have it, or move. Don't try to underbid your competitor. That is a game two can play. Don't think that he underbid you, for possibly your argument was an education that only needed his confirmation to make the sale.

Don't be jealous of your competitor, for that is the surest flattery. Don't underestimate him, for he surely must have good points or he could not hold so many friends and patrons as he does. Don't overestimate him, for he probably makes as many mistakes as you, and also probably no more. The mote and beam are probably reversed according to the end of the line you look at it.

Don't think you can work for every patient, for just as people affect you—some attract, others repel—so do you affect them. It is the personal equation, and you can't control it.

It is the work that you do that counts. It does not help nor hinder whether the other fellow is busy or idle. Attend to your own business and make as few mistakes as should be allotted to you as a human being.

Join your dental society and take an active part. It will help you, and you will help it.—*Western Dental Journal.*

